# Is a specialist breathlessness service more effective and cost-effective for patients with advanced cancer and their carers than standard care? Findings of a mixed-method randomised controlled trial

**DOI:** 10.1186/s12916-014-0194-2

**Published:** 2014-10-31

**Authors:** Morag C Farquhar, A Toby Prevost, Paul McCrone, Barbara Brafman-Price, Allison Bentley, Irene J Higginson, Chris Todd, Sara Booth

**Affiliations:** Primary Care Unit, Department of Public Health and Primary Care, University of Cambridge, Institute of Public Health, Robinson Way, Cambridge, CB2 0SR UK; School of Nursing, Midwifery & Social Work, University of Manchester, Jean McFarlane Building, Oxford Rd, Manchester, M13 9PL UK; Department of Primary Care and Public Health Sciences, Division of Health and Social Care Research, School of Medicine, King’s College London, 7th Floor Capital House, 42 Weston Street, London, SE1 3QD UK; Institute of Psychiatry, King’s College London, De Crespigny Park, London, SE5 8AF UK; Formerly: Palliative Care Team, Cambridge University Hospitals’ NHS Foundation Trust, Addenbrooke’s Hospital, Hills Rd, Cambridge, CB2 0QQ UK; Department of Palliative Care, Policy & Rehabilitation, King’s College London, Cicely Saunders Institute, Denmark Hill, London, SE5 9PJ UK; Palliative Care Team, Cambridge University Hospitals’ NHS Foundation Trust, Addenbrooke’s Hospital, Hills Rd, Cambridge, CB2 0QQ UK

**Keywords:** Breathlessness, Cancer, Advanced disease, Randomised controlled trial, Complex intervention, Mixed methods

## Abstract

**Background:**

Breathlessness is common in advanced cancer. The Breathlessness Intervention Service (BIS) is a multi-disciplinary complex intervention theoretically underpinned by a palliative care approach, utilising evidence-based non-pharmacological and pharmacological interventions to support patients with advanced disease. We sought to establish whether BIS was more effective, and cost-effective, for patients with advanced cancer and their carers than standard care.

**Methods:**

A single-centre Phase III fast-track single-blind mixed-method randomised controlled trial (RCT) of BIS versus standard care was conducted. Participants were randomised to one of two groups (randomly permuted blocks). A total of 67 patients referred to BIS were randomised (intervention arm n = 35; control arm n = 32 received BIS after a two-week wait); 54 completed to the key outcome measurement. The primary outcome measure was a 0 to 10 numerical rating scale for patient distress due to breathlessness at two-weeks. Secondary outcomes were evaluated using the Chronic Respiratory Questionnaire, Hospital Anxiety and Depression Scale, Client Services Receipt Inventory, EQ-5D and topic-guided interviews.

**Results:**

BIS reduced patient distress due to breathlessness (primary outcome: −1.29; 95% CI −2.57 to −0.005; *P* = 0.049) significantly more than the control group; 94% of respondents reported a positive impact (51/53). BIS reduced fear and worry, and increased confidence in managing breathlessness. Patients and carers consistently identified specific and repeatable aspects of the BIS model and interventions that helped. How interventions were delivered was important. BIS legitimised breathlessness and increased knowledge whilst making patients and carers feel ‘not alone’. BIS had a 66% likelihood of better outcomes in terms of reduced distress due to breathlessness at lower health/social care costs than standard care (81% with informal care costs included).

**Conclusions:**

BIS appears to be more effective and cost-effective in advanced cancer than standard care.

**Trial registration:**

RCT registration at ClinicalTrials.gov NCT00678405 (May 2008) and Current Controlled Trials ISRCTN04119516 (December 2008).

**Electronic supplementary material:**

The online version of this article (doi:10.1186/s12916-014-0194-2) contains supplementary material, which is available to authorized users.

## Background

Breathlessness (dyspnoea) is a common distressing symptom of advanced cancer, impacting physically, emotionally and socially on patients and families [1]. Occurring in 90% of lung cancer and 50% to 70% of all cancers, its prevalence increases rapidly towards the end of life [2].

The experience of breathlessness is complex [3]. Given its multifactorial causes and multidimensional impacts [2], and absence of a single effective palliative treatment, complex interventions are indicated. Early intervention models used non-pharmacological single-disciplinary approaches [4-6]. More recent models are multi-disciplinary [7], utilising evidence-based pharmacological [8-10] and non-pharmacological [11-13] component interventions [14,15]. Few have been evaluated with randomised controlled trial (RCT) methodology.

The Breathlessness Intervention Service (BIS) is a multi-disciplinary complex intervention combining non-pharmacological and pharmacological interventions to support breathless patients with advanced disease, theoretically underpinned by a palliative care approach [16-18]. Developed and evaluated [1,19-22] using the Medical Research Council (MRC) framework for complex interventions [23], it has undergone a Phase III RCT with two sub-protocols: one for advanced cancer and one for advanced non-malignant disease (differing service model for each) [24]. This paper reports the findings of the sub-protocol for advanced cancer in relation to the following research questions:

1. Is BIS more effective than standard care for patients with intractable breathlessness from advanced malignant disease?
2. Does it reduce patient and carer distress due to breathlessness and increase patients’ sense of mastery of the symptom?
3. What are the experiences and views of those who use BIS (patients and their informal carers)?
4. Is BIS cost-effective?

## Methods

A detailed study protocol [24] and detailed intervention description [16,17] are published elsewhere. Box 1 outlines the two-week intervention for advanced cancer (intervention duration determined by disease trajectory). The BIS team comprises: a palliative care medical consultant (with dedicated clinical sessions and a research interest in breathlessness), a clinical specialist occupational therapist (lead clinician for the service), a clinical specialist physiotherapist and an administrator. Each professional contributes their individual strengths and skills in particular areas, but all are able to deliver the core interventions outlined in Box 1, using a psychologically-informed approach. At a weekly multidisciplinary team meeting cases are allocated to the most appropriate professional based on information derived from the referral; many patients receive visits from at least two professionals on the team. The intervention is delivered predominantly in the home-setting with visits typically lasting 1 to 1.5 hours. Visits include interventions relevant to that person (outlined in Box 1) and formulation of an individually-tailored exercise plan, for example, walking incrementally increasing distances in their local environment using a handheld fan to manage breathlessness (the first attempt would be accompanied by a member of the BIS team). Further details can also be found on the BIS website [18].

Standard care was defined as specialist outpatient appointments in secondary care (for example, oncology) which may include specialist nurse input, and primary care services. Key aspects of study design, sampling, outcome measures, data collection and analysis for the advanced cancer sub-protocol are outlined below.

### Study design

We recruited patients with advanced cancer referred to BIS into a Phase III mixed-method single-blind pragmatic fast-track (waiting list) RCT of BIS versus standard care (November 2008 to January 2012). Ethical approval was given by by Cambridgeshire 2 NHS REC (Ref:08/H0308/157); RCT registration at ClinicalTrials.gov NCT00678405 and Current Controlled Trials ISRCTN04119516.

### Randomisation and blinding

Participants were randomised to one of two groups using randomly permuted blocks of random size two, four and six, generated by the study statistician and concealed within sealed opaque envelopes until allocation notification by the intervention deliverer. The fast-track (intervention) group received BIS immediately; the waiting-list (control) group received BIS after two-weeks. All participants received standard, including palliative, care. Data collection-design facilitated researcher-blinding to group allocation for the collection of primary and key secondary outcomes at the key measurement point, that is, planned unblinding occurred during the two-week follow up interview (t3) only after collection of this outcome data and prior to qualitative data collection about the intervention.

### Participants

Consecutive cancer patients referred to BIS (from primary or secondary care) were invited to participate by letter. Patients were eligible if they met BIS referral criteria (that is, diagnosed appropriately-treated cause of breathlessness, troubled by breathlessness in spite of optimisation of underlying illness, and might benefit from a self-management programme) and excluded if they had received BIS previously. Recruited patients were asked to identify who gave them the most help and support at home and these informal carers were also invited to participate. All participating patients and informal carers gave informed consent. Patients who were unwilling to participate in the trial continued to have access to BIS.

### Sample size

A sample size of 60 randomised patients (26 analysed per arm, allowing for dropout) provided 80% power to detect a 2-point difference in mean distress at two-weeks between groups (SD = 2.5, alpha = 5%), with increased precision anticipated from adjustment for baseline.

### Baseline and outcome measures

Patient distress due to breathlessness [4,5] (the primary outcome on which the trial was powered) was measured using a numerical rating scale (NRS). Other key patient-reported variables included disease-specific health related quality of life (Chronic Respiratory Questionnaire: CRQ [25]), and anxiety and depression (Hospital Anxiety and Depression Scale: HADS [26]). Key carer-reported outcome measures included an NRS for carer distress due to patient breathlessness and HADS. A generic health status measure (EQ-5D [27]) and measure of service use (Client Services Receipt Inventory (CSRI) [28]) were administered for health economic analyses. Brief qualitative topic-guided interviews were also conducted with all patients and carers to explore expectations and experiences of BIS. All outcomes were participant-reported; the full outcomes list is reported elsewhere [24].

### Data collection

Participating patients and carers completed a baseline interview (t1: week 1) before randomisation. These mixed-method interviews included the quantitative patient- and carer-reported measures and qualitative interviews described above (carers were interviewed separately where possible). A two-week follow up interview (t3: week 3) was designed to represent completion of BIS for the intervention arm, or end of the waiting-list period prior to BIS for controls. A final interview (t5: week 5) was conducted four-weeks from baseline; this represented two-weeks after BIS for the intervention arm and completion of BIS for controls. All interviews were conducted in home-settings.

### Analysis

Complete case intention-to-treat analyses were conducted using a linear regression model; each outcome was adjusted for its baseline. A 5% level of statistical significance was used. Costs were calculated combining service use data (CSRI) for eight-weeks and two-weeks prior to baseline and t3, respectively, with UK 2011/2012 unit costs [29]. Informal care (unpaid hours/week from family/friends performing specific tasks) was valued at average UK wages (£11.21/hour) [30]. Costs of BIS visits were estimated at £91 (based on specialist nurse contacts which averaged the rehabilitation specialists’ wages) and phone contacts at one-quarter of this. Costs were compared using regression models, controlling for baseline, and using bootstrap methods with 1,000 resamples to address the likely skewed distribution. Sensitivity analyses were performed around these key costs by increasing/decreasing costs by 25% and 50%. Costs, with and without informal care, were combined with the primary outcome and EQ-5D-derived quality-adjusted life years (QALYs), with uncertainty explored using cost-effectiveness planes [31]. Cost-effectiveness planes are a graphical way of comparing one intervention to another or to usual care. The vertical axis of the plane shows the extra costs incurred by an intervention while the horizontal axis shows the extra outcomes achieved; both of these could be positive or negative resulting in four quadrants on the plane. Bootstrap methods can then be used to produce a large number of cost and outcome combinations which can be plotted on the plane to show the probability of the intervention resulting in higher costs and better outcomes, higher costs and worse outcomes, lower costs and better outcomes or lower costs and worse outcomes.

Qualitative interview data were transcribed and anonymised. Two analytic approaches were taken to the comprehensive qualitative dataset. First, transcripts of all post-intervention interviews (fast-track group t3s and control t5s; n = 53, no qualitative interview for one patient) were categorised into one of three intervention impact levels by three analysts working independently (Level 1: Significant impact - clearly stated BIS made a difference; Level 2: Some impact - no major change recognised, but valued specific aspects of BIS; Level 3: No impact – BIS made no difference at all). Categorisation commenced with a small number of interviews. Analysts then compared categorisations, discussing and resolving differences, clarifying level-definitions and data interpretation, before repeating for all remaining interviews.

Second, as qualitative analysis of this sized dataset (n = 53) would be unmanageable, 20 patient (and associated carer) intervention arm transcripts were purposefully sampled against a novel stratified four-cell matrix of primary outcome changes by t3, to achieve a maximum diversity sample [32]. The four cells represented: (Cell 1) patients who improved most on primary outcome (who, predictably, had high baseline scores; Biggest Improvers); (Cell 2) patients with high baseline scores (to match Cell 1) but who improved least (Limited Improvers); (Cell 3) patients who worsened (who transpired to have low-middling baseline scores; Worseners); and (Cell 4) patients with closest match to Cell 3 baseline scores but who improved most (Moderate Improvers). Anonymised interview transcripts for this purposive sample were imported into NVivo software [33], to facilitate framework analysis [34]. This principally descriptive analysis explored the nature of BIS impacts, which aspects were valued, and possible mechanisms of impact.

## Results

**Figure 1:**
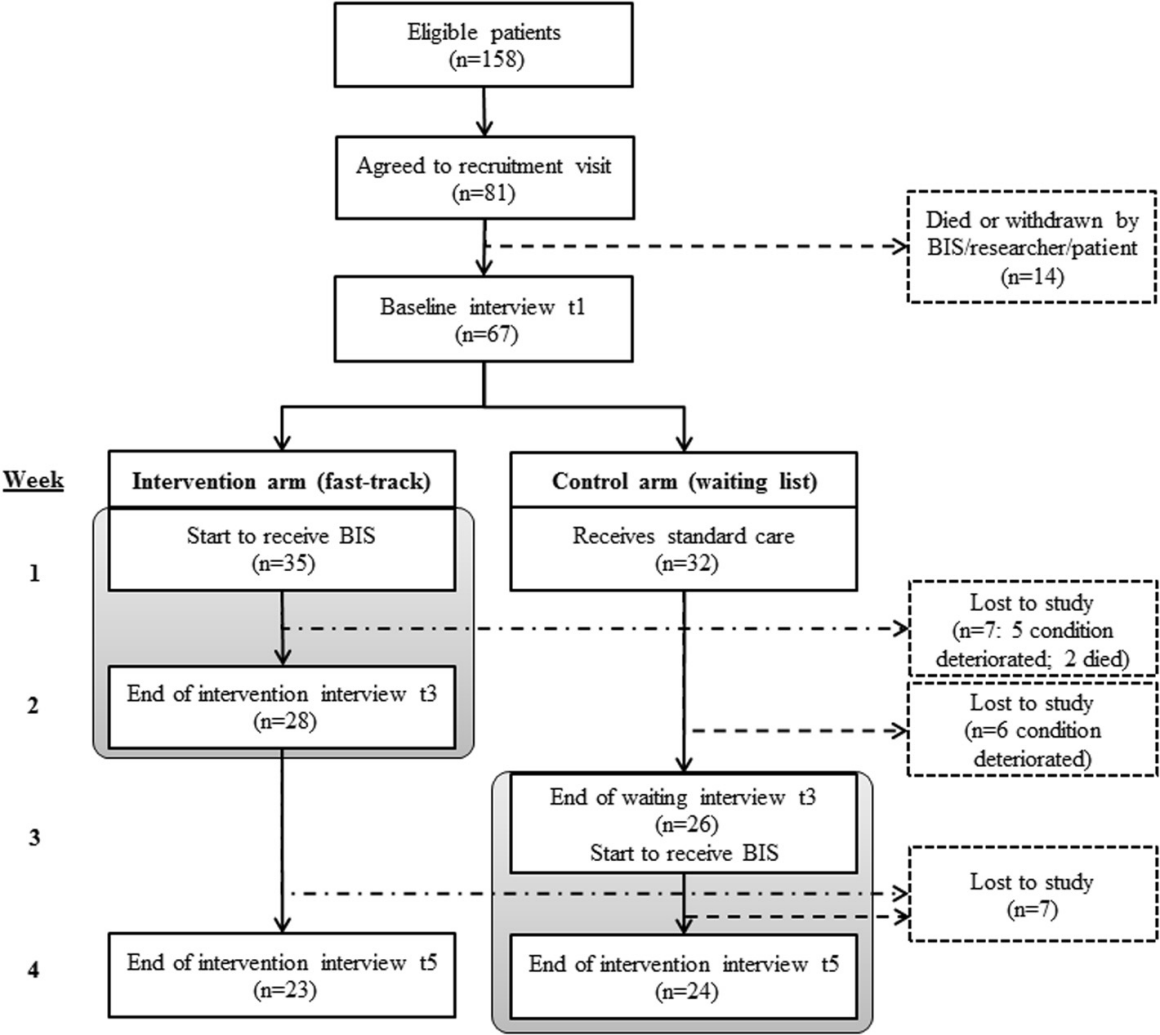
**CONSORT flow diagram.**

Figure 1 (CONSORT diagram) illustrates randomisation of 67 patients; 54 completed the trial to key outcome measurement (t3). Thirteen patients withdrew due to deterioration prior to t3: seven and six from intervention and control arms, respectively, (including two intervention arm deaths), with very similar mean baseline distress due to breathlessness scores of 5.43 for the intervention arm withdrawals and 5.86 for the control arm withdrawals. The intent was for researchers to remain blinded to group allocation until after measurement of the primary outcome at t3; this was achieved for 52% (28/54) of the patients. The remainder unintentionally unblinded researchers earlier in the t3 interview either by inadvertently mentioning contact with BIS or by BIS resources (for example, information sheets) being visible to the researcher in the patient’s home.

Baseline characteristics were well matched across trial arms (Table 1). Patients were predominantly older, female and living with others; lung was the commonest cancer site, then breast. Patients’ mean level of anxiety (7.52) was higher by one-third of a standard deviation compared with the mean population norm (mean population norm for anxiety 6.14; SD = 3.76) [35] and their mean level of depression (6.26) was notably higher (mean population norm for depression 3.68; SD = 3.07) by 0.84 SD of the norm distribution. Just under half had clinically-meaningful anxiety scores and about a quarter clinically-meaningful depression scores. Breathlessness, performance status and co-morbidity were as anticipated. Patient ‘distress due to breathlessness’ and CRQ domain-scores were similar across trial arms.

**Table 1:** **Baseline characteristics of patients and carers by arm, BIS Phase IIIm**

**Baseline characteristics**	**Mean (SD) or % (number)**		
	**Intervention arm**	**Control arm**	**Total**
**Patient baseline characteristics**			
Age (years)	70 (9.4)	67 (13.3)	69 (11.5)
Sex (female)	59% (21)	62% (20)	61% (41)
Lives alone	34% (12)	28% (9)	31% (21)
Cancer site (primary):			
Lung	45% (16)	54% (17)	49% (33)
Breast	25% (9)	13% (4)	19% (13)
Rectal/bowel	6% (2)	6% (2)	6% (4)
Prostate	6% (2)	3% (1)	4% (3)
Lymphoma	3% (1)	6% (2)	4% (3)
Mesothelioma	3% (1)	6% (2)	4% (3)
Gastro-oesophageal junction	3% (1)	3% (1)	3% (2)
Renal	3% (1)	3% (1)	3% (2)
Endometrial	0% (0)	3% (1)	2% (1)
Hepatocellular	3% (1)	0% (0)	2% (1)
Bladder	0% (0)	3% (1)	2% (1)
Unknown primary	3% (1)	0% (0)	2% (1)
Charlson Co-Morbidity Index (0 to 9)^a^	6.34 (2.30)	5.63 (2.14)	6.00 (2.24)
Australia-modified Karnofsky Performance Scale (0 to 100)^b,c^	71.1 (12.6)	74.1 (14.8)	72.5 (13.6)
Modified Borg: breathlessness at rest (0 to 10)^a^	1.69 (1.09)	1.39 (1.77)	1.55 (1.45)
Modified Borg: breathlessness on exertion (0 to 10)^a^	5.27 (2.18)	5.13 (2.21)	5.20 (2.18)
NRS worst breathlessness 24 hours (0 to 10)^a^	4.97 (2.46)	4.19 (2.28)	4.60 (2.39)
NRS breathlessness now (0 to 10)^a^	2.29 (1.74)	1.81 (1.38)	2.06 (1.59)
NRS average breathlessness 24 hours (0 to 10)^a^	3.83 (1.67)	3.38 (1.93)	3.61 (1.80)
NRS distress due to breathlessness (0 to 10)^a^	5.17 (2.81)	4.94 (2.84)	5.06 (2.81)
Anxiety score (HADS) (0 to 21)^a^	6.97 (4.01)	8.09 (3.31)	7.52 (3.70)
Depression score (HADS) (0 to 21)^a^	6.61 (2.73)	5.91 (2.99)	6.26 (2.87)
Anxiety (HADS)^a^:			
Normal (0 to 7)	55% (18)	53% (17)	54% (35)
Mild (8 to 10; possible clinical disorder)	27% (9)	19% (6)	23% (15)
Moderate (11 to 14; probable clinical disorder)	15% (5)	25% (8)	20% (13)
Severe (15 to 21; probable clinical disorder)	3% (1)	3% (1)	3% (2)
Depression (HADS)^a^:			
Normal (0 to 7)	70% (23)	78% (25)	74% (48)
Mild (8 to 10; possible clinical disorder)	21% (7)	9% (3)	15% (10)
Moderate (11 to 14; probable clinical disorder)	9% (3)	13% (4)	11% (7)
Severe (15 to 21; probable clinical disorder)	0% (0)	0% (0)	0% (0)
CRQ dyspnoea score (1 to 7)^b^	3.42 (0.99)	3.39 (0.98)	3.41 (0.98)
CRQ fatigue score (1 to 7)^b^	3.38 (1.33)	3.20 (1.07)	3.29 (1.20)
CRQ emotional function score (1 to 7)^b^	4.43 (1.02)	4.46 (1.30)	4.35 (1.06)
CRQ mastery score (1 to 7)^b^	4.57 (1.15)	4.46 (1.30)	4.52 (1.22)
**Number of respondents (patients)**	**33-35**	**32**	**65-67**
**Carer baseline characteristics**			
Carer age (years)	65.6 (13.4)	63.5 (12.2)	64.6 (12.7)
Carer sex (female)	70% (14)	67% (14)	68% (28)
Carer employment status:			
Employed – full time	11% (2)	24% (5)	18% (7)
Employed – part time	22% (4)	5% (1)	13% (5)
Retired	61% (11)	57% (12)	59% (23)
Other (e.g. unemployed due to illness/student)	6% (1)	14% (3)	10% (4)
NRS carer distress due to patient breathlessness (0 to 10)^a^	3.56 (2.44)	3.86 (2.82)	3.72 (2.62)
Carer anxiety score (HADS) (0 to 21)^a^	7.89 (5.33)	6.76 (3.59)	7.28 (4.45)
Carer depression score (HADS) (0 to 21)^a^	3.39 (3.91)	4.29 (2.93)	3.87 (3.40)
Carer anxiety (HADS)^a^:			
Normal (0 to 7)	44% (8)	57% (12)	51% (20)
Mild (8 to 10; possible clinical disorder)	17% (3)	24% (5)	21% (8)
Moderate (11 to 14; probable clinical disorder)	28% (5)	19% (4)	23% (9)
Severe (15 to 21; probable clinical disorder)	11% (2)	0% (0)	5% (2)
Carer depression (HADS)^a^:			
Normal (0 to 7)	78% (14)	76% (16)	77% (30)
Mild (8 to 10; possible clinical disorder)	22% (4)	19% (4)	20% (8)
Moderate (11 to 14; probable clinical disorder)	0% (0)	5% (1)	3% (1)
Severe (15 to 21; probable clinical disorder)	0% (0)	0% (0)	0% (0)
**Number of respondents (carers)**	**18-20**	**19-21**	**39-41**
No carer/no carer interview	43% (15)	34% (11)	39% (26)

^a^High score is worse; ^b^high score is better; ^c^a score of 70 represents ‘cares for self yet unable to carry on normal activity or to do active work’. BIS, Breathlessness Intervention Service; CRQ, Chronic Respiratory Questionnaire; HADS, Hospital Anxiety and Depression Score; NRS, numerical rating scale.

Carers were predominantly older women, and about a third were employed. Carers’ mean level of anxiety was moderately higher than population norms by 0.30 SD of the norm distribution [35] and depression was comparable (higher by 0.06 SD). About half had clinically-meaningful anxiety scores and just under a quarter had clinically-meaningful depression scores. Carer ‘distress due to patient breathlessness’ was lower than patients’ , but similar across trial arms.

### Changes in patient distress due to breathlessness

Comparison of change in patient distress due to breathlessness (primary outcome; NRS range 0 to 10) by the key outcome measurement point (t3) showed that patients randomised to the intervention arm achieved a significantly greater, 1.68-point, reduction compared with 0.23-point reduction for controls: adjusted difference of −1.29 (95% CI: −2.57 to −0.005), *P* = 0.049 (Table 2). There was little change in carer distress.

**Table 2:** **Changes in patient distress due to breathlessness (primary outcome), mastery of breathlessness, anxiety and depression, by trial arm**

**Trial arms and outcomes**	**t1** ^**a**^ **Mean (SD)**	**t3 Mean (SD)**	**t5 Mean (SD)**	**Difference in mean t3 adjusted for baseline (I minus C)**	**With 95% ** **confidence interval**	***P*** **-value**
Control arm (waiting-list)	Baseline	Completed control	Completed intervention			
Intervention arm (fast-track)	Baseline	Completed intervention	Post-intervention			
**Primary outcome: NRS distress due to breathlessness (0 to 10)** ^**b**^						
Control arm (waiting-list)	4.65 (2.99)	4.42 (3.01)	2.58 (1.82)	−1.29	(−2.57, -0.005)	*P* = 0.049
Intervention arm (fast-track)	5.11 (2.78)	3.43 (2.95)	3.65 (3.07)			
**Key secondary outcomes:**						
**CRQ** ^**c**^ **Mastery (1 to 7)**						
Control arm (waiting-list)	4.71 (1.27)	4.72 (1.21)	4.72 (1.12)	0.20	(−0.35, 0.76)	*P* = 0.47
Intervention arm (fast-track)	4.53 (1.13)	4.81 (1.29)	4.72 (1.18)			
**HADS** ^**b**^ **anxiety (0 to 21)**						
Control arm (waiting-list)	7.88 (3.41)	7.85 (3.59)	7.61 (3.96)	0.017	(−1.52, 1.56)	*P* = 0.98
Intervention arm (fast-track)	7.00 (4.08)	7.07 (5.05)	6.77 (3.52)			
**HADS** ^**b**^ **depression (0 to 21)**						
Control arm (waiting-list)	5.88 (2.94)	6.23 (2.89)	6.13 (2.80)	−0.30	(−1.79, 1.20)	*P* = 0.69
Intervention arm (fast-track)	6.38 (2.17)	6.22 (3.36)	6.27 (3.12)			
No. of respondents	52-54	53-54	45-47			

^a^For those with a t3 score; ^b^high score is worse; ^c^high score is better. CRQ, Chronic Respiratory Questionnaire; HADS, Hospital Anxiety and Depression Score; NRS, numerical rating scale; SD, standard deviation.

### Change in mastery of breathlessness, anxiety and depression

Mean CRQ mastery scores improved only negligibly by t3 on the intervention arm and remained stable for controls (not statistically significant; Table 2). No significant differences were found between trial arms to t3 on other CRQ domains (dyspnoea, fatigue or emotional function). Mean anxiety scores (HADS) remained fairly stable to t3 (both arms). Mean depression scores decreased slightly by t3 in the intervention arm, increasing slightly for controls (not statistically significant). There was little change in other patient or carer outcomes.

### Reported benefit of BIS

Categorisation of qualitative interviews inferred that for 68% (n = 36) of patients, or patient-carer dyads, BIS had had a significant impact (Level 1). A further 28% (n = 15) indicated BIS had had some impact (that is, no major change, but they valued specific aspects of BIS; Level 2) and 4% (n = 2) reported no impact (Level 3).

Table 3 shows the Impact Categorisation Levels for the purposively-sampled qualitative interviews, sampled for maximum diversity of change on primary outcome to t3. Given the skewed distribution of the levels it is unsurprising that most were Level 1 and 2, even among primary outcome ‘Worseners’ (Cell 3).

**Table 3:** **Purposively sampled t3 qualitative interviews (intervention arm) and their Impact Categorisation Levels**

**Change in patient NRS distress due to breathlessness** ^**a**^ **(primary outcome) t1 to t3 (and Impact Categorisation Level)**	
**Cell 1: Biggest Improvers (from high baseline scores)**	**Cell 2: Limited Improvers (high baseline score Cell 1 matches who improved the least)**
533: NRS distress reduced from 8–1 (Level 2)	530: NRS distress reduced from 10–8 (Level 1)
564: NRS distress reduced from 7–1 (Level 1)	591: NRS distress: reduced from 10–8 (Level 1)
583: NRS distress reduced from 9–3 (Level 1)	624: NRS distress reduced from 10–8 (Level 2)
648: NRS distress reduced from 7–1 (Level 1)	536: NRS distress reduced from 5–3 (Level 1)
603: NRS distress reduced from 5–0 (Level 1)	630: NRS distress unchanged 4–4 (Level 2)
**Cell 3: Worseners (who turned out to have a low-middling baseline scores)**	**Cell 4: Moderate Improvers (closest baseline score Cell 3 matches who improved the most)**
563: NRS distress increased from 4–9 (Level 2)	501: NRS distress reduced from 4–1 (Level 1)
578: NRS distress increased from 3–7 (Level 2)	637: NRS distress reduced from 3–0 (Level 3)
569: NRS distress increased from 5–7 (Level 1)	616: NRS distress reduced from 3–1 (Level 1)
612: NRS distress increased from 5–7 (Level 1)	658: NRS distress reduced from 8–4 (Level 2)
559: NRS distress increased from 5–6 (Level 2)	587: NRS distress reduced from 9–5 (Level 1)

^a^High score is worse. Level 1 = significant impact; Level 2 = some impact; Level 3 = no impact. NRS, numerial rating score. Three-digit numbers = participant identity numbers.

Qualitative analysis of the purposively-sampled interviews identified both the nature of BIS impacts and which aspects of the BIS model were valued, as well as the possible mechanisms of impact. Impacts described included patients and carers noting reductions in fear, anxiety, worry and feelings of panic, and greater confidence about breathlessness. Patients reported managing their breathlessness better, and some carers living separately from patients reported that the latter contacted them less.

Patients and carers valued specific aspects of the BIS model. The multi-disciplinary staff expertise was repeatedly noted: their knowledge about breathlessness, and strategies suggested to manage it, made clear they understood difficulties of life with breathlessness. Participants described particularly helpful characteristics of BIS staff, such as being relaxed and easy to talk to, listening and reassuring. They valued the time BIS gave them to talk about breathlessness with an expert. Being seen at home was especially helpful, as was the positive ‘can do’ approach of BIS and unexpected attention given to carers.

Patients and carers identified interventions delivered by BIS that were therapeutic including: providing and teaching use of a handheld fan; encouragement of exercise (including pedometer provision/goal-setting); coaching in breathing techniques, positioning, pacing and relaxation (including provision of BIS’ mindfulness-based body-scan CD and teaching visualisation techniques); occupational therapy aids; information and education (verbal, printed sheets and hand-drawn diagrams) - learning that ‘being breathless won’t kill me’ was cited as particularly liberating. Other valued actions included medication changes, referral-on to other services (for example, hospice day care) and advising on daily strategies to ease breathlessness (often described as ‘lots of little things’).

Explanatory analysis suggests it was not only the *provision* of these interventions that was important: *how* they were delivered was key to their impact. The data suggest they were delivered through the provision of knowledge, with specialist expertise, which increased patients’ and carers’ confidence. For example, some reported receiving handheld fans from other clinicians but identified the way BIS delivered this intervention as different: BIS explained how and when to use the fan and how it might work, so legitimising what at first appeared an unlikely effective intervention.

Thus, the mechanism of impact appeared to relate to improved knowledge, enhancing patients’ and carers’ understanding and their confidence in living with the symptom. BIS acknowledged, and thereby validated, breathlessness; patients and carers reported no longer feeling alone. Box 2 provides illustrative quotes for gaining knowledge and confidence, and some participant-identified interventions.

Reviewing transcripts for the categorisation exercise identified that more than half of patients had further contacts planned with BIS beyond the key measurement of the primary outcome: 52% (28/54) described planned contacts beyond t3. Thus, any improvement in the primary outcome at t3 was early impact and there may have been further benefit beyond this.

### Costs and cost-effectiveness

Table 4 shows contacts with services (face-to-face or telephone) and associated costs. At baseline most patients received hospital, general practitioner and informal care. Costs for some individual services were quite different between arms, but (in common with most other economic evaluations) zero costs were common and standard deviations high. Total costs at baseline were higher in the intervention arm. Between baseline and t3 more than half received hospital care (both arms) and fewer in the intervention group used inpatient or informal care (Table 4). Costs during follow-up were notably lower, mainly due to the shorter cost-period compared to baseline. Adjusting for baseline costs, the intervention group had health/social care costs on average £211 less than controls (95% CI, −£918 to £310), decreasing to £182 and £154 when intervention costs were increased by 25% and 50%, respectively (intervention remained dominant). Total costs (including informal care) were £354 less for the intervention group (95% CI, −£1020 to £246).

**Table 4:** **Service use and cost in eight-weeks prior to baseline assessment and between baseline and t3**

**Service use and cost during the eight-weeks prior to baseline assessment**						
**Formal and informal care**	**Intervention arm (number = 35)**			**Control arm (number = 32)**		
**Service**	**Number (%)**	**Mean (SD) contacts**	**Mean (SD) cost**	**Number (%)**	**Mean (SD) contacts**	**Mean (SD) cost**
Inpatient	15 (43)	13.0 (12.1)	3187 (5807)	16 (50)	8.1 (5.5)	2306 (3208)
Other hospital care	32 (91)	5.9 (6.3)	712 (827)	31 (97)	4.5 (2.9)	566 (377)
GP	31 (89)	3.2 (2.3)	145 (165)	28 (88)	3.5 (1.9)	134 (102)
Nurse	27 (77)	10.4 (19.0)	278 (605)	26 (81)	2.5 (1.9)	63 (72)
Other health professionals	17 (49)	2.0 (1.1)	42 (59)	15 (47)	1.9 (1.2)	33 (55)
Social care	5 (14)	29.6 (30.3)	101 (343)	5 (16)	24.8 (48.8)	110 (462)
Total formal care costs			4464 (6017)			3212 (3434)
Informal care	32 (91)	20.4 (18.8)	1673 (1690)	32 (100)	25.1 (25.1)	2249 (2248)
Total costs			6137 (6099)			5461 (4393)
**Service use and cost between baseline and t3**						
	**Intervention arm (number = 28)**			**Control arm (number = 26)**		
**Service**	**Number (%)**	**Mean (SD) contacts**	**Mean (SD) cost**	**Number (%)**	**Mean (SD) contacts**	**Mean (SD) cost**
BIS intervention	27 (96)	1.9 (2.0)	119 (62)	2 (8)	1.5 (0.7)	5 (20)
Inpatient	2 (7)	3.0 (2.8)	123 (547)	3 (12)	6.3 (6.8)	418 (1614)
Other hospital care	15 (54)	1.5 (0.8)	105 (115)	14 (54)	1.4 (0.6)	96 (107)
GP	10 (36)	1.2 (0.6)	22 (38)	13 (50)	1.3 (0.5)	27 (32)
Nurse	11 (39)	3.0 (3.8)	37 (96)	12 (46)	1.8 (1.6)	25 (46)
Other health professionals	5 (18)	1.2 (0.4)	11 (28)	3 (12)	1.0 (0.0)	3 (9)
Social care	4 (14)	4.3 (6.5)	20 (59)	3 (12)	15.7 (22.9)	45 (190)
Total formal care costs			436 (604)			618 (1627)
Informal care	22 (79)	20.3 (20.8)	358 (452)	25 (96)	23.4 (25.2)	504 (564)
Total costs			794 (866)			1121 (1635)

Costs in 2011/2012 £s. BIS, Breathlessness Intervention Service; GP, general practitioner; SD standard deviation.

Lower health/social care costs and better primary outcome results for the intervention arm indicated dominance over the control condition. Cost-effectiveness planes showed a 66.4% likelihood that the intervention would have *lower* health/social care costs and *better* outcomes in terms of reduced distress due to breathlessness than the control condition and a 30.9% likelihood of *higher* health/social care costs and *better* outcomes (likelihood of lower costs and worse outcomes 1.4%; likelihood of higher costs and worse outcomes 1.3%). The figures for total costs (including informal care) were 80.9% and 16.4%, respectively (likelihood of lower costs and worse outcomes 2.2%; likelihood of higher costs and worse outcomes 0.5%). The maximum QALY-gain over a two-week period was 0.038. The intervention resulted in an incremental QALY-gain of 0.0002 (95% CI, −0.001 to 0.002). While technically indicating dominance for the intervention, the difference was small and variation substantial. This is reflected in the cost-effectiveness plane showing BIS having a 40.5% likelihood of *lower* health/social care costs and a *greater* QALY-gain than the control condition, a 21.4% likelihood of *higher* costs and a *greater* QALY-gain, a 27.3% likelihood of *lower* costs and a *smaller* QALY-gain and a 10.8% likelihood of *higher* costs and a *smaller* QALY-gain. With informal care included the figures were 50.9%, 11.0%, 32.2% and 5.9%, respectively. The cost-effectiveness planes are available as supplementary material [see Additional file 1: Figures S1 to S4].

## Discussion

In this Phase III RCT, BIS reduced distress due to breathlessness in patients with advanced cancer significantly more than the control group. When costs were linked to the primary outcome, the findings indicate BIS was cost-effective over the follow-up period; however, this was less apparent when using QALYs, possibly reflecting the relatively short follow-up period. The reasonably sized effect is a useful indicator of the impact of BIS and was the primary outcome, but the qualitative data were more informative for service evaluation. BIS reportedly made a positive difference to 94% of patients and carers with impacts including reduced fear and worry, and increased confidence about breathlessness. Patients and carers consistently noted specific, identifiable and repeatable aspects of the BIS model and a range of interventions they found helpful in reducing the impact of breathlessness, many of which suggest modulation of central perception [15,36]. Although a list of interventions the participants found beneficial is useful, the way these interventions were delivered was important. BIS appears to work by legitimising breathlessness and increasing knowledge and confidence whilst making patients and carers feel ‘not alone’. Henoch *et al*. [3], identified the importance of ‘coping capacity’ in helping lung cancer patients manage the palliative phase; BIS may be increasing this.

Although we identified modest group-level reduction in distress due to breathlessness, distress decreased considerably for some individuals but increased for others – some increase may be anticipated in progressive disease. As Table 2 demonstrated, these ‘Worseners’ did, however, continue to report benefit from BIS. This raises the question of whether the selected quantitative primary outcome was the most appropriate to measure accurately the effect of the intervention. Further, any measured impact was early impact as many had further input from BIS beyond the key measurement point; plus potential added effects of BIS referrals-on to other services is unknown.

We believe this is the first fast-track RCT in advanced cancer to attempt to use single-blinding and in this we were partially successful. Additionally, although trials increasingly include qualitative elements where appropriate [37], this mixed-method trial was unusual in collecting qualitative data on all participants, not just a sub-sample. This enabled quantification of aspects of the qualitative data and purposive sampling on changes in the primary outcome.

The trial suffered slow recruitment due to slower referrals of cancer patients and a higher refusal rate than anticipated. Thirteen patients were lost to follow up but were evenly spread across trial arms, with very similar mean baseline scores, and no data at an intermediate time-point with which to further assess sensitivity of the results to the assumptions of the primary analysis approach. The main result was borderline statistically significant; sensitivity analysis to the handling of missing data would inevitably cast some doubt on the main result; therefore, this is a limitation. The trial might have benefitted from later timing of the key measurement point to include more BIS contacts in the analysis of impact suggesting that, in some cases, the BIS intervention may require slightly longer than two-weeks to be delivered for patients with advanced cancer (reflecting the intervention’s individualised nature). Longer-term follow up would have enabled examination of maintenance of benefit.

This trial supports positive findings of earlier studies of intervention services for breathlessness in advanced cancer [4-6,38]. The key differences between these and BIS are that BIS is multidisciplinary, delivered in patients’ homes and takes a flexible individualised approach to the number and content of contacts. Recent work by Ellis *et al*. [39], suggests this may explain the lower attrition experienced by BIS than other services. In addition, BIS was more robustly developed and evaluated due to the availability of the MRC framework for complex interventions.

## Conclusions

In conclusion, BIS appears to be more clinically-effective, and cost-effective, for patients with breathlessness in advanced cancer and their carers than standard care.

## Box 1: Service model for Breathlessness Intervention Service (BIS) for patients with advanced cancer

The Breathlessness Intervention Service (BIS) is a multi-disciplinary complex intervention combining non-pharmacological and pharmacological interventions to support breathless patients with advanced disease, theoretically underpinned by a palliative care approach. As such, it is a flexible intervention, responsive to need, but with a minimum set of core components. Consultations usually occur in the patient’s own home.

**Core components for patients with advanced cancer:**

**BIS team:** Palliative Care Medical Consultant; Clinical Specialist Occupational Therapist; Clinical Specialist Physiotherapist

**Medical assessment:** All reviewed at Multi-Disciplinary Team (therefore all have MD review) but seen by doctor only if complex medical problems or intractable medical/psychosocial problems

**First appointment:** Maximum wait of one week for first appointment

**Range of face-to-face visits:** one to four

**Range of telephone contacts (with patient/primary care staff):** four to six

**Average length of service contact:** two weeks

**Service outcome measures collected at first assessment:** Numerical Rating Scale (NRS) for breathlessness, emotion and confidence at first and last assessment; Physiological measures, for example, oxygen saturation, heart rate

**Non-pharmacological interventions:** Most patients seeing service are mobile and breathless and, therefore, most patients have non-pharmacological interventions

**Pharmacological interventions:** As indicated after review by doctor

**First stage interventions (selection and application as clinically indicated):**

Selective use and application of these interventions: explanation and reassurance; hand-held fan; breathing control; activity pacing and exercise; anxiety management; psychological support; information fact sheets; emergency plan; positioning to reduce work of breathing (rest, recovery and activity); education to patient, carer and health care generalists; lifestyle adjustment; individualised exercise plan; relaxation and visualisation; airway clearance techniques; advice regarding nutrition and hydration; support to family and patient to utilise education and selfsupport programmes; sleep hygiene; brief cognitive therapy; pharmacological review; well-being intervention; formal relaxation therapy; mindfulness CD; referral to specialist services (see below)

**Second stage interventions:**

Second stage interventions likely to be applied concurrently with first stage interventions: pharmacological review, for example, low dose opioids, antidepressants, anxiolytics; referral to specialist services (see below); referral for LTOT or SBOT assessment; acupuncture

**Other symptom management:** Frequently required

**Documentation:** individualised patient plan; detailed letter to patient of record of consultations with all BIS clinicians; discharge summary to referrer with copies to GP, specialist services the patient was already in contact with (for example, respiratory physicians), other involved health care professionals (for example, district nurses, nursing home care staff); supplementary medical letters more common

Referrals: Palliative care specialist service (note: rapid access available); Community therapists; Hospice day services; Pulmonary rehabilitation; other specialist assessment; community groups; charitable and self-help groups, for example, British Legion, Age UK, Macmillan

[LTOT, long term oxygen therapy; SBOT, short burst oxygen therapy]

## Box 2: Illustrative quotes from purposive sample about mechanisms of impact and valued interventions

**Mechanisms of impact**

**Mechanism of impact - gaining knowledge:**

Patient: ‘she said to me put my lips … like that … and [breathe] through my mouth. I thought […] ‘how is that going to work?’ […] but I must be honest, it’s brilliant. Do you know it helps more than doing it through your nose? […] well I was very interested, because I went to the bathroom and of course when I got back I couldn’t breathe […] and I thought ‘well give it go’, you know, and … […] it does help, it really does. […] I thought the nose and the mouth was the most important thing for you to do, but her telling me that and another thing as well, when I get out of breath, is to put my hand on my tummy … *puff puff puff* … and do that, and you know, it’s amazing really, it sounds so pathetic when you say something … It is simple, it’s not a thing you’d think of doing [putting] your hands on your tummy and do that, you wouldn’t … […] She was really helpful’

[530t3pc; Impact Categorisation Level 1 – Significant impact; Cell 2 – Low Improver on primary outcome]

**Mechanism of impact - feeling not alone:**

Carer: ‘It’s nice to know it’s there, that if I’ve got any problems or worries that I can phone up and say ‘I think he’s a lot, lot, lot worse today, what can I do?’ … you know? […] Because you know … we’re a long way from [hospital], and you can’t just sort of keep popping up and going there because it exhausts him anyway … and it’s nice to know there’s someone at the end of the phone to say ‘well try this, try this, and if it doesn’t get any better do this …”

[569t3c; Impact Categorisation Level 1 – Significant impact; Cell 3 – Worsener on primary outcome]

**Mechanism of impact - gaining confidence:**

Patient: ‘I’ve gained more confidence and like I say, with that fan, [I] went upstairs […] and whereas I normally sit on the bed and I’ve got a cylinder of oxygen under the bed, I might have got that … but I got myself round with just the fan, because she said ‘quite often you think you’re worse than what you are, but if you had a bit of air in your face’ and […] that did work’

Interviewer: ‘And what did you find most helpful about the Service so far?’

Patient: ‘Probably the little tips she’s given me really and build your confidence up’

[536t3pc; Impact Categorisation Level 1 – Significant impact; Cell 2 – Low Improver on primary outcome]

**Valued interventions**

**Valued intervention - handheld fan:**

Patient: ‘…she was really helpful. I mean she’s given me exercises to do, she’s left me a fan … and she’s told me how to use that, she’s shown my husband and me how to deal with [it]. […] if I get breathless the fan will help … I breathe in and blow out … and blow out […]. I was surprised really because I’ve used these handheld fans in the summer in the car before I got this … and I always thought they were just to cool you down! But I must admit … when she showed me how to use it, at the time, I found it was very good’

[603t3pc; Impact Categorisation Level 1 – Significant impact; Cell 1 – Big Improver on primary outcome]

**Valued intervention - exercise:**

Patient: ‘[She] suggested exercising my legs, sort of holding them up like that […] to try and strengthen my thigh muscles […] I can sit here and do what she suggested and it’s certainly easier to climb the stairs’

[578t3p; Impact Categorisation Level 2 – Some impact; Cell 3 – Worsener on primary outcome]

**Valued intervention - pacing:**

Patient: ‘She was saying if you want to do something think about doing it, don’t just get up and do it, […] because I get up and do it suddenly I suddenly get breathless and I can’t do it, and one of the ideas she had was well, if you think about doing something, think of what the best way of doing it is which isn’t going to make you breathless and then go about it that way and see what the result is. And I tried that, so instead of thinking … I want something from the garage … I’ll go and get it, I think ‘I want something from the garage, right, I’ll go down there in a minute and pick it up and sort of get my body working’, and then go and do it, and that doesn’t stress you as much. Odd little things like that’

[587t3p; Impact Categorisation Level 1 – Significant impact; Cell 4 – Moderate Improver on primary outcome]

**Valued intervention–‘breathlessness won’t kill me’:**

Interviewer: ‘what was the most helpful thing [they] did from your point of view?’

Patient: ‘[Telling me that] breathlessness is not harmful. […] because I thought getting out of breath wasn’t good for you. It’s not good for you, but it’s not going to harm you […] And that reassures you, because […] you think ‘I’m not going to die, this is just a blip, you’ll get over it’ […] and now I know what to do, so she was really helpful. I wouldn’t have known that if I hadn’t seen her, I would have just gone on thinking ‘oh dear …’ […] and the panic you see makes you feel worse. If you’re panicked about something it makes your breathing worse because you breathe different when you panic. Even you would if you was in a panic, you would be breathing different. […] and I don’t get that any more. […] I know how to deal with it [now]’

[603t3pc; Impact Categorisation Level 1 – Significant impact; Cell 1 – Big Improver on primary outcome]

## Additional file

Additional file 1: Figure S1. Cost-effectiveness plane based on health/social care costs and reduction in patient distress due to breathlessness. **Figure S2.** Cost-effectiveness plane based on total costs and reduction in patient distress due to breathlessness.** Figure S3.** Cost-effectiveness plane based on health/social care costs and QALYs. **Figure S4.** Cost-effectiveness plane based on total costs and QALYs.

## Abbreviations

BIS: Breathlessness Intervention Service
CI: confidence interval
CRQ: Chronic Respiratory Questionnaire
CSRI: Client Services Receipt Inventory
HADS: Hospital Anxiety and Depression Scale
MRC: Medical Research Council
NHS: National Health Service
NRS: numerical rating scale
QALY: quality-adjusted life year
RCT: randomised controlled trial
REC: research ethics committee
SD: standard deviation
