# Supplementary material for: Is a specialist breathlessness service more effective and cost-effective for patients with advanced cancer and their carers than standard care? Findings of a mixed-method randomised controlled trial
Source: BMC Med. 2014 Oct 31;12:194. doi: 10.1186/s12916-014-0194-2 (PMC4222435; doi:10.1186/s12916-014-0194-2)
Supplement: Additional file 1: Figure S1. — Cost-effectiveness plane based on health/social care costs and reduction in patient distress due to breathlessness. Figure S2. Cost-effectiveness plane based on total costs and reduction in patient distress due to breathlessness. Figure S3. Cost-effectiveness plane based on health/social care costs and QALYs. Figure S4. Cost-effectiveness plane based on total costs and QALYs. [file 12916_2014_194_MOESM1_ESM.docx]

**Supplementary Figure 1. Cost-effectiveness plane based on health/social care costs and reduction in patient distress due to breathlessness.**

**Supplementary Figure 2. Cost-effectiveness plane based on total costs and reduction in patient distress due to breathlessness.**

**Supplementary Figure 3. Cost-effectiveness plane based on health/social care costs and QALYs.**

**Supplementary Figure 4. Cost-effectiveness plane based on total costs and QALYs.**
